# An update on liver surgery – a new terminology and modern techniques

**DOI:** 10.1515/iss-2023-0032

**Published:** 2024-01-04

**Authors:** Verena Tripke, Nils Sommer

**Affiliations:** Department of General, Visceral and Transplantation Surgery, University Medical Center of the Johannes Gutenberg University, Mainz, Germany; Department of General, Visceral, Thoracic and Vascular Surgery, University Hospital Bonn, Bonn, Germany; Chirurgische Arbeitsgemeinschaft Junge Chirurgie CAJC, Deutsche Gesellschaft für Allgemein- und Viszeralchirurgie, Berlin, Germany

**Keywords:** liver surgery, 3D imaging, minimally invasive surgery, liver tumor, classification of liver resections

## Abstract

Liver surgery is the cornerstone of the curative treatment of malignant liver tumors. However, the liver anatomy is very complex, and liver surgery is still associated with relevant morbidity despite many technical advances. The Brisbane nomenclature is used worldwide to classify liver resection. However, this nomenclature has several limitations as multiple terms are used for the same type of resection. Non-anatomical resections, multiple resections, and combined bilio-vascular resections were not mentioned. Therefore, new terminologies have been proposed for the precise and simple classification of liver resection. Furthermore, in recent years, many technical innovations have been introduced in liver surgery, such as 3D imaging systems and indocyanine green fluorescence, for better preoperative and intraoperative identification of tumor localization and critical vascular structures. Minimally invasive techniques are used more frequently in liver surgery. Potential benefits include less intraoperative blood loss, less pain, and a shorter hospital stay. The implementation of robotic systems also has an impact on liver surgery, and the number of cases reported in the literature is constantly increasing. The potential benefits of robotic liver resection over laparoscopic liver resection will be the subject of future studies.

## Introduction

Liver surgery is the cornerstone in the treatment of liver malignancies, as complete tumor removal offers a chance to cure the patient. Due to technical and medical innovations, as well as improved complication management, the postoperative morbidity and mortality rates were significantly reduced. However, liver surgery remains challenging because liver anatomy is very complex and variable. Precise knowledge of tumor localization and its relationship to important vascular structures is necessary. The Brisbane nomenclature for hepatic anatomy and resection is used worldwide to describe the type of resection. Usually, preoperative imaging modalities, such as MRI and/or CT scans, are used for planning the surgical strategy and determining resectability. Intraoperative sonography and palpation were used to localize the tumor and surrounding vessels. In recent years, many technical innovations have been introduced in liver surgery, particular 3D-imaging and -navigation systems, as well as indocyanine green fluorescence (ICG). 3D-models allow better visualization of the tumor and the surrounding vessels during the process of preoperative planning, while 3D-navigation and ICG intraoperatively support the surgeon during tumor resection

Furthermore, minimally invasive surgery is increasingly used for liver resection. Laparoscopic liver surgery has already shown benefits, such as less intraoperative blood loss, less postoperative pain, and a shorter hospital stay than open liver surgery. The robotic approach was also introduced to liver surgery, and the number of publications addressing robotic liver surgery is constantly increasing. However, the role of robotic liver surgery remains unclear, and there is still no evidence of the superiority of the robotic approach over the conventional laparoscopic approach.

This review aims to provide a short update on the recent advances and innovations in the field of liver surgery consisting of 10 interesting papers of the last two years.

## New terminologies

**Figure 1:**
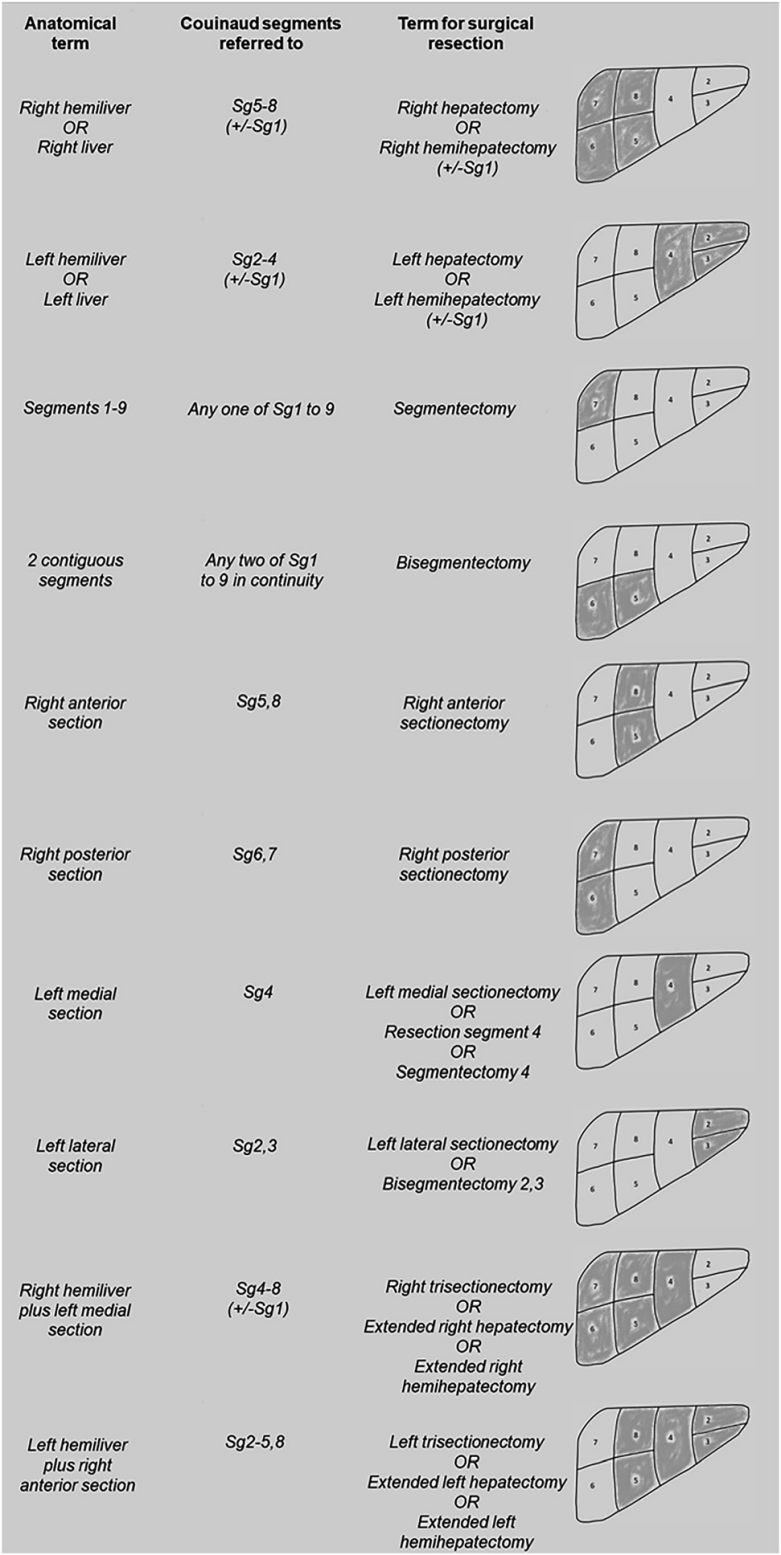
Brisbane 2000 terminology of liver anatomy and resections based on Couinaud’s segments [11].

In 2000, the Brisbane nomenclature of hepatic anatomy and resections was approved at the biennial meeting of the International Heptao-Pancreato-Biliary Association in Brisbane and has been used worldwide to classify liver resections (Figure 1). However, the Brisbane nomenclature has several limitations as multiple terms are used for the same type of resection, for example “right trisectionectomy,” “extended right hepatectomy” and “extended right hemihepatectomy,” and non-anatomical resections as well as multiple resections and combined bilio-vascular resections are not mentioned. Therefore, Nagino et al. proposed a new terminology for hepatectomy in 2021 [1]: The “New World” Terminology bases on the Couinaud hepatic segmental anatomy just like the Brisbane nomenclature. The letter “H” indicates hepatectomy. Non-anatomical resections are named according to the segment number and apostrophe (’). For *en bloc* resection of multiple segments, the number of resected segments is mentioned in ascending order, while separate resections of multiple segments are separated by a slash (/). Finally, the number of non-anatomically resected segments is mentioned. The add-ons “-B,” “-PV,” “-HA,” “-RHV,” “-MHV” or “-IVC” express the combined resection of the extrahepatic bile duct, portal vein, hepatic artery, right hepatic vein, middle hepatic vein or inferior vena cava, respectively. The authors did not include subsegment resections and lymphadenectomy to the nomenclature to keep it simple. Examples of the “New World” Terminology for hepatectomy are shown in Table 1.

**Table 1:** Examples of the “New World” terminology.

Number	Traditional nomenclature	New terminology
1	Nonanatomical resection of segment 7	H7′
2	Anterior sectionectomy	H58
3	Nonanatomical separate resection of segment 5 and 6	H5′/6′
4	Nonanatomical en bloc resection of segment 5 and 6	H5′6′
5	Left lateral sectionectomy and 2 separate nonanatomical resections of segment 6 and 8	H23/6′/8′
6	Left trisectionectomy	H23458
7	Right hemihepatectomy	H5678
8	Right trisectionectomy with resection of caudate lobe	H145678
9	Posterior sectionectomy with partial extension to segment 8 and resection of right hepatic vein	H678′-RHV
10	2 nonanatomical separate resections of segment 4	H4′/4′
11	Left hemihepatectomy with resection of caudate lobe and extrahepatic bile duct	H1234-B
12	Right trisectionectomy with resection of caudate lobe, extrahepatic bile duct and portal vein	H145678-B-PV
13	Right trisectionectomy with resection of caudate lobe, extrahepatic bile duct and inferior vena cava	H145678-B-IVC
14	Right hemihepatectomy with part. extension to segment 4, resection of caudate lobe, extrahepatic bile duct and middle hepatic vein	H156784′-B-MHV
15	Left trisectionectomy with resection of caudate lobe, extrahepatic bile duct, portal vein and hepatic artery	H123458-B-PV-HA

H, hepatectomy; B, extrahepatic bile duct resection; PV, portal vein resection; IVC, inferior vena cava resection; MHV, middle hepatic vein resection; RHV, right hepatic vein resection; HA, hepatic artery resection.

Another update to the Brisbane 2000 terminology proposed by Wakabayashi et al. in 2022 is the Tokyo 2020 terminology for liver anatomy and resections [2]. The authors presented seven definitions and five recommendations for segmentectomy. First, the authors defined anatomical liver resection as complete removal of the liver parenchyma confined within the responsible territory of the portal vein. Second, anatomical segmentectomy is defined as the complete removal of the territories of the third-order portal venous branches of the Couinaud segment. The third definition defines anatomical subsegmentectomy as the removal of the liver parenchyma within portal territories of less than a Couinaud segment, which are also defined as cone units. The authors described a subsegment as an anatomical portion of a Couinaud segment, which is defined as a cone unit based on the subsegmental inflow. Furthermore, segment 4 of is redefined as two subsegments (segment 4a apical and segment 4b basal). The definition of segment 9 from the Brisbane 2000 terminology was abandoned, and the caudate lobe was defined based on the portal ramifications. Segment 1 is divided into three anatomical parts: the Spiegel, paracaval, and caudate processes. After redefining the liver anatomy, the authors make several recommendations: Firstly, “segmentectomy” and “subsegmentectomy” should not be used for non-anatomical resections. Second, there are two main approaches to the responsible Glissonean pedicle, either from the hilum or the liver surface, to perform segmentectomies or subsegmentectomies. Furthermore, the authors recommend using color dye or indocyanine green dye for the visualization of the territory of the responsible portal vein to perform precise segmentectomy or subsegmentectomy. Finally, following the appropriate intersegmental/sectional plane, which contains hepatic veins along the plane or crossing the planes, is suggested as the key to performing precise anatomical liver resection.

## 3D-imaging

Due to the complexity and high variability of liver anatomy, precise knowledge of tumor localization to the adjacent vascular structures is necessary for planning liver resections. This is a challenging task even for experienced liver surgeons. In recent years, technological advances in the field of 3D-imaging made it possible the transfer of CT or MRI scans of the liver into a 3D model, which aids in preoperative planning.

Lopez-Lopez et al. conducted a multicenter retrospective study from seven centers that used a 3D preoperative planning system for patients with perihilar cholangiocarcinoma (pCCA) [3]. 41 patients with pCCA were evaluated using 3D modelling software. Four patients were considered inoperable based on the 3D model. Of the 37 patients scheduled to surgery five patients were inoperable. A questionnaire was used to record the surgeons’ experience. A better understanding of tumor size, extension, and vascular relationship, as well as an improved interpretation of the vascular and biliary anatomy, is described.

Moreover, 3D printing is increasingly used for the planning of liver resections. Huber et al. described their experience with 3D printing in 10 cases of complex liver surgery [4]. The printed models included the parenchyma, hepatic veins, vena cava, portal vein, hepatic artery, and tumors. Vascular reconstruction was performed in seven cases, and R0 resection was achieved in nine patients. 3D printing was considered beneficial for intraoperative orientation and visualization of the critical areas of vascular reconstruction. A literature review by Liu et al. reported an increasingly frequent use of 3D printing for complex liver resections [5]. Visualization of the anatomy and diameter of blood vessels and bile ducts, as well as their relationship to tumors, are described as beneficial. In addition, the surgeon obtains a precise idea about the total and residual liver volumes. Another publication from Saito et al. confirmed the usefulness of 3D printing in terms of vessel anatomy and the relationship between vascular structures and tumors, as well as the parenchymal cutting plane [6]. In addition, 3D printing offers several benefits for medical education. However, the high costs and complex creation processes may be disadvantageous. A meta-analysis by Jiang et al. included 16 studies (randomized controlled trials or cohort studies) comparing preoperative 3D reconstruction with conventional CT scans in patients undergoing hepatectomy for primary hepatic carcinoma [7]. This study showed improved operation time and reduced intraoperative blood loss when preoperative 3D reconstruction was performed. The length of hospital stay and complication rate were not significantly affected.

Another technology used to improve the visualization of important anatomical structures is the intraoperative utilization of indocyanine green fluorescence (ICG). ICG has various applications in liver surgery [8]. First, ICG allows visualization of the bile ducts during liver resection and helps identify structures that need to be saved. A recent consensus meeting stated that the use of ICG improved bile duct identification and reduced the incidence of iatrogenic injuries. ICG can also help identify the tumor intraoperatively when administered 1–2 weeks before surgery. Furthermore, ICG can be administered intraoperatively to identify portal territories when performing anatomical liver resection. Especially, in laparoscopic liver surgery this technique is widely used to overcome the limitations of laparoscopic liver resection.

## Minimally invasive liver surgery

Over the past few decades, laparoscopic techniques have been increasingly used for liver surgery. However, benchmarks for laparoscopic liver resection (LLR) are lacking. In 2022, the International Robotic and Laparoscopic Liver Resection Study Group aimed to establish relevant intraoperative and postoperative benchmark values for LLR in low-risk patients [9]. Three types of procedures (laparoscopic left lateral sectionectomy (LLS), left hepatectomy (LH), and right hepatectomy (RH)) were selected from three difficulty groups (low, intermediate, and high difficulty) to reduce the heterogeneity of the procedures. Only pure laparoscopic resections were analyzed from an international multicenter database of 11,893 patients. Patients with a tumor diameter ≥10 cm or Child–Pugh B liver cirrhosis were excluded from the study. In total, 3098 LLR met the inclusion criteria and 15 benchmark values for short-term perioperative outcomes were established. The benchmarks for duration of operation and conversion rate after LLS, LH, and RH were 209.5, 302, and 426 min; 2.1 , 13.4, and 13.0 %, respectively, and for blood loss ≥500 mL and blood transfusion rate 3.2 , 20, 47.1; and 0, 7.1, and 10.5 %, respectively. Furthermore, the benchmarks for postoperative morbidity, major morbidity (>Clavien–Dindo grade II), and 90-day mortality after LLS, LH, and RH were 11.1 , 20, and 50; 0, 7.1, and 20; and 0, 0, and 0 %, respectively. This study aimed to provide an up-to-date reference for the “best achievable” results for LLR.

Similar to the laparoscopic technique, the robotic approach has been introduced for liver surgery. Recently, an increasing number of robotic liver resections have been reported. A recent review by Bozkurt et al. summarized the current evidence regarding robotic liver surgery [10]. The widely proven advantages of minimally invasive liver surgery are less intraoperative blood loss, less postoperative pain, and shorter hospital stay than open liver surgery. Whether the robotic approach is superior to conventional LLR remains a matter of discussion. However, no standardized and safe technique for robotic liver resection has been reported in the literature. Nevertheless, robotic techniques are reported as safe and feasible. Moreover, the robotic approach offers the same advantages as the conventional laparoscopic approach when compared with open liver surgery. In complex cases, robotic liver resection seems to offer a small benefit concerning intraoperative blood loss and a lower conversion rate compared to LLR owing to a wider range of motion and integrated 3D imaging system. However, the robotic system has also several disadvantages. Currently reported systems do not provide feedback on tissue tension, and their cost is very high. Nevertheless, the cost will soon decrease and new robotic surgery systems will be launched in the market. With further technical innovations, such as navigation systems and artificial intelligence technologies, the advantages of robotic systems in liver surgery might increase.

## Conclusions

There are many new developments in liver surgery. New terminology for liver anatomy and resections simplifies the nomenclature of liver resections and will be used more frequently in the literature. However, the new nomenclature might confuse surgeons not specialized in liver surgery and especially physicians of other medical disciplines. Therefore, it will be important to routinely use the new terminology in operation reports and publications eventually in addition to the Brisbane 2000 nomenclature.

3D imaging provides important additional information for the attending surgeon, such as a better understanding of tumor size, extension, and vascular relationship, as well as an improved interpretation of the vascular and biliary anatomy. 3D printing is increasingly used in cases of complex liver resection for better visualization of the vasculature. However, randomized controlled trials are required to prove the benefits of preoperative 3D imaging. ICG is a widely accepted tool for better intraoperative visualization of the bile ducts, tumor localization, and identification of portal territories. In addition to conventional laparoscopic liver resection, a robotic approach has been introduced for liver surgery. There might be minor advantages concerning the conversion rate and intraoperative blood loss in complex liver resections. However, as the costs are relatively high, and no standardized technique for robotic liver resection has been reported in the literature, the role of robotic liver surgery remains unclear and will have to be investigated in further studies.
